# Fast and Furious: Ethylene-Triggered Changes in the Metabolism of Papaya Fruit During Ripening

**DOI:** 10.3389/fpls.2019.00535

**Published:** 2019-04-26

**Authors:** João Paulo Fabi, Samira Bernardino Ramos do Prado

**Affiliations:** ^1^ Department of Food Science and Experimental Nutrition, School of Pharmaceutical Sciences, University of São Paulo, São Paulo, Brazil; ^2^ Food Research Center (FoRC), CEPID-FAPESP (Research, Innovation and Dissemination Centers, São Paulo Research Foundation), São Paulo, Brazil; ^3^ Food and Nutrition Research Center (NAPAN), University of São Paulo, São Paulo, Brazil

**Keywords:** papaya, climacteric fruits, ethylene, fruit metabolism, cell wall, fruit quality

## Abstract

Papaya is a climacteric fleshy fruit characterized by fast ripening after harvest. During the relatively short postharvest period, papaya fruit undergoes several changes in metabolism that result in pulp softening and sweetening, as well as the development of a characteristic aroma. Since papaya is one of the most cultivated and appreciated tropical fruit crops worldwide, extensive research has been conducted to not only understand the formation of the quality and nutritional attributes of ripe fruit but also to develop methods for controlling the ripening process. However, most strategies to postpone papaya ripening, and therefore to increase shelf life, have failed to maintain fruit quality. Ethylene blockage precludes carotenoid biosynthesis, while cold storage can induce chilling injury and negatively affect the volatile profile of papaya. As a climacteric fruit, the fast ripening of papaya is triggered by ethylene biosynthesis. The generation of the climacteric ethylene positive feedback loop is elicited by the expression of a specific transcription factor that leads to an up-regulation of 1-*aminocyclopropane-1-carboxylic acid* (ACC) *synthase* (ACS) and *ACC-oxidase* (ACO) expression, triggering the system II ethylene biosynthesis. The ethylene burst occurs about 3 to 4 days after harvest and induces pectinase expression. The disassembling of the papaya cell wall appears to help in fruit sweetness, while glucose and fructose are also produced by acidic invertases. The increase in ethylene production also results in carotenoid accumulation due to the induction of cyclases and hydroxylases, leading to yellow and red/orange-colored pulp phenotypes. Moreover, the production of volatile terpene linalool, an important biological marker for papaya’s sensorial quality, is also induced by ethylene. All these mentioned processes are related to papaya’s sensorial and nutritional quality. We describe the understanding of ethylene-triggered events that influence papaya quality and nutritional traits, as these characteristics are a consequence of an accelerated metabolism during fruit ripening.

## Introduction

Papaya (*Carica papaya* L.) is a typical climacteric fleshy fruit that is appreciated worldwide because of the sweetness and characteristic flavor of its soft yellow or orange/red pulp (Fabi et al., 2007, 2010b). Tropical countries from Asia are the main producers of papaya, accounting for 56% of worldwide production. However, countries from South America (16%), Africa (10%), and Central America (9%) are also important producers of papaya (Food and Agriculture Organization of the United Nations (FAOSTAT), 2017). As papayas have a relatively short shelf life compared to other fruits, maintaining fruit quality during transport from producing countries to consumer centers (e.g., USA and Europe) is a challenge. In 2016, the main countries that produced papayas for exportation were Mexico (47%), Guatemala (14%), and Brazil (11%, Food and Agriculture Organization of the United Nations (FAOSTAT), 2017), with Mexico being the main supplier to the United States and Brazil the main supplier to Europe (Evans and Ballen, 2012).

European recommendations for papaya exporting countries take into account fruit softening as a determinant factor in fruit shelf life (CBI Ministry of Foreign Affairs, 2018), since the fast softening during papaya ripening facilitates physical injury during handling and transportation. Thus, as the susceptibility of papayas to disease increases proportionally with softening (Manrique and Lajolo, 2004), the recommendation for exportation is to maintain the fruit at 10°C during shipping to prevent overripening due to heat (CBI Ministry of Foreign Affairs, 2018). However, as will be discussed later, low temperatures negatively impact some fruit quality attributes of ripe papayas.

The ripening of fleshy fruits is a physiological process that alters appearance, texture, flavor, and aroma. These changes function to attract seed-dispersing organisms (Giovannoni, 2004). In climacteric fruit, such as tomatoes, bananas, and papayas, the onset of ripening coincides with an increase in respiration and ethylene production, the latter being essential to induce molecular mechanisms responsible for accelerating senescence and for the physiological changes that occur during ripening (Abeles et al., 1992; Gapper et al., 2013). The ripening process in climacteric fruits induces changes in both sensorial and nutritional qualities that are essential for consumer acceptability. Some climacteric fruits are harvested unripe and treated with exogenous ethylene or ethylene-derived molecules to precipitate ripening. Thus, ethylene appears to be the main hormone responsible for regulating the molecular pathways that influence the development of the sensorial and nutritional attributes of climacteric fruits (Lü et al., 2018). It has long been known that the safe and effective control of ethylene-mediated responses could extend the postharvest shelf life of climacteric fruits (Wills et al., 1981). However, interfering with natural ethylene-mediated responses during ripening could also negatively impact fruit quality.

While the mechanism by which ethylene is involved in fruit ripening has been thoroughly studied, efforts are still needed to fully understand this process. The ethylene burst in climacteric fruit is controlled by an autocatalytic mechanism, named system II, that synthesizes ethylene (McMurchie et al., 1972; McManus, 2012). Ethylene synthesis involves the conversion of S-adenosyl methionine (SAM) to 1-aminocyclopropane-1-carboxylic acid (ACC) by the action of 1-amino cyclopropane-1-carboxylic acid synthase (ACS), in which ACC is converted to ethylene by ACC oxidase (ACO) (Yang and Hoffman, 1984). ACS and ACO enzymes have already been identified in papayas, and their responses are increased with ethylene production and reduced when ethylene is blocked (Razali et al., 2013). A decrease in ACS and ACO occurs in papayas stored at low temperatures, but levels are restored after exogenous ethylene treatment (Zou et al., 2014). The ethylene downstream cascade involves multiple transcription factors, including ethylene response factors (ERFs), that are involved in the control of plant growth, defense, responses to the environment, and plant hormones (Xie et al., 2016), including those involved in the papaya ripening process (Li et al., 2013). Transcription factors of the MADS-box, NAC, and AP2/ERF gene families are also involved in the control of papaya ripening (Fabi et al., 2012). More recently, a NAC transcription factor, rather than MADS transcription factors, was found to regulate ACS and ACO expression during papaya ripening (Lü et al., 2018). Papaya has not undergone whole-genome duplication, unlike other climacteric fruits where this process has been utilized to duplicate the MADS transcription factors that form the ripening circuits (Périn et al., 2002; Lee et al., 2013). NAC is one of the largest plant-specific transcription factor families, with members involved in many developmental processes such as senescence, stress, cell wall formation, and embryo development (Lü et al., 2018). Lü et al. (2018) have suggested that instead of neofunctionalization of the duplicated MADS genes, plants without whole-genome duplication may have repurposed their carpel senescence NAC to generate a positive feedback loop where ethylene regulates ripening, as is the case with papayas. They also suggested that ethylene generated by this feedback loop is autocatalytic. A NAC transcription factor expressed in climacteric fruits, such as papayas and peaches, binds to the promoter regions of some of the key ripening-related genes stimulating their expression in pigment accumulation, volatile secondary metabolite production, cell wall softening, and sugar accumulation (Lü et al., 2018).

Therefore, the ethylene-mediated effects in fruit metabolism that influence the softening, sweetness, flavor, and color of papaya pulp during ripening will be further discussed.

## Pulp Softening is the Main Biochemical Modification that Occurs During Papaya Ripening

In climacteric fleshy fruits, researches and producers give special attention to ethylene-induced textural changes during ripening, as changes in peel and pulp not only influence softening, crispness, and juiciness (Chaïb et al., 2007) but also increase postharvest losses (Manrique and Lajolo, 2004). In fact, textural changes in most of the fleshy fruits result from complex mechanisms that primarily influence plant cell wall architecture, whose breakdown is considered as the major factor responsible for the pulp-softening process (Brummell, 2006).

The cell wall architecture of fleshy fruits is comprised of complex polysaccharides, such as pectin, hemicellulose, and cellulose, as well as minor components including proteins and phenolic compounds (Carpita and Gibeaut, 1993). Cellulose is comprised of long, rigid, and inextensible microfibrils of 1,4-β-d-glucose (Glc) residues, which are bound tightly together by hydrogen bonds (Brummell, 2006). Hemicelluloses represent a diverse range of structural polymers that constitute the plant cell wall within fruit pulp (Scheller and Ulvskov, 2010). In dicotyledonous plants, such as papayas, xyloglucan (XYL) is the major hemicellulose (Tucker et al., 2017). As with cellulose, XYL consists of a backbone of 1,4-β-d-Glc residues such as cellulose, but smaller and substituted with 1-6-α-d-xylose (Xyl) side chains. Furthermore, these Xyl side chains can be substituted at the O-2 position with β-galactose (Gal) or α-arabionse (Ara; Scheller and Ulvskov, 2010). Pectin is a complex and heterogeneous polysaccharide that is mainly comprised of α-1,4-d-galacturonic acid (GalA) residues that have varying degrees of acetyl and methyl esterification, and these residues are called homogalacturonan (HG). Xylosylation may further modify HG into xylogalacturonans (XGs). Pectin also contains structures made up of repeating units of intercalated GalA (1,4-α-d-GalpA) and rhamnose (1,2-α-l-Rhap) called rhamnogalacturonan type I (RG-I). These structures have side groups of arabinose (arabinan), galactose (galactan), and type I arabinogalactan at the O-4 position of the Rha residues (Mohnen, 2008; Maxwell et al., 2012). Rhamnogalacturonan type II (RG-II) structures are less common in papayas and are composed of HG molecules with side groups of up to 13 different sugars and more than 20 types of glycosidic linkages (Mohnen, 2008; Bar-Peled et al., 2012). The firmness of fleshy fruits results from turgor pressure maintenance by the cell wall while also maintaining cellular adhesion (Wang et al., 2018). Pulp softening occurs by the water dissolution of the majority of these polysaccharides from the primary cell wall and middle lamella, with pectin being the main one (Brummell and Harpster, 2001).

Structural changes that occur in the cell wall during ripening are regulated by hydrolases responsible for degrading cell wall polysaccharides (Gapper et al., 2013; Balic et al., 2014), whose expression is generally regulated by ethylene production (Tucker et al., 2017). Fruit softening is a complex event that involves several enzymes including pectinases and hemicellulases; however, pectinases, such as polygalacturonases (PGs), pectate lyases (PLs), and pectin methyl esterases (PMEs) appear to be the major enzymes that act on fleshy fruit softening. Polygalacturonases remove the galacturosyl residues from pectin (Atkinson et al., 1998), PLs cleave de-esterified pectin (Marín-Rodríguez et al., 2002), and PMEs hydrolyze methyl-groups of esterified polyuronides (Wakabayashi et al., 2003). Furthermore, side chains of pectin can be degraded by other glycosidases, such as β-galactosidases, which remove the galactosyl residues from pectin and from XYL (Smith et al., 2014); α-arabinofuranosidases, which remove arabinosyl from pectin (Sozzi et al., 2002; Itai et al., 2003); and rhamnogalacturonases, which remove α-1,2 linkages between galacturonosyl and rhamnosyl residues (Wong, 2008).

Despite multiple glycoside hydrolases seeming to be responsible for papaya softening, the main enzymes that play a central role in pulp softening are the PGs (Fabi et al., 2014). Some contribution of hemicellulose degradation to pulp softening appears to occur as an increase in endoxylanase expression occurs during papaya ripening (Huerta-Ocampo et al., 2012). Furthermore, β-galactanases are also related to papaya pulp softening through the hydrolysis of both the pectic and the hemicellulosic fractions (Lazan et al., 2004; Fabi et al., 2014). In order to understand the role of ethylene in the expression of cell wall-degrading enzymes, researchers have treated papayas with 1-methylcyclopropene (1-MCP), an ethylene antagonist. As expected, this had a strong effect on pulp softening (Fabi et al., 2007). The pulp firmness of 1-MCP-treated papayas decreased marginally during ripening, although not enough to reach an edible state, and there was no detectable PG activity. Notably, 1-MCP-treated papayas were unable to soften at the same rate as untreated papayas (Fabi et al., 2007, 2009; Sañudo-Barajas et al., 2009). Treatment with 1-MCP also reduced endoxylanase protein levels (Huerta-Ocampo et al., 2012).

To confirm that ethylene affects PG activity and, therefore, pulp softening during papaya ripening, Fabi et al. (2009) found that treatment with exogenous ethylene had induced PG expression with a concomitant increase in pulp softening. Furthermore, agroinfiltration of PG1 in 1-MCP-treated papayas significantly enhanced pulp softening compared with 1-MCP-treated papayas that were agroinfiltrated with an empty vector (Fabi et al., 2014).

**Figure 1:**
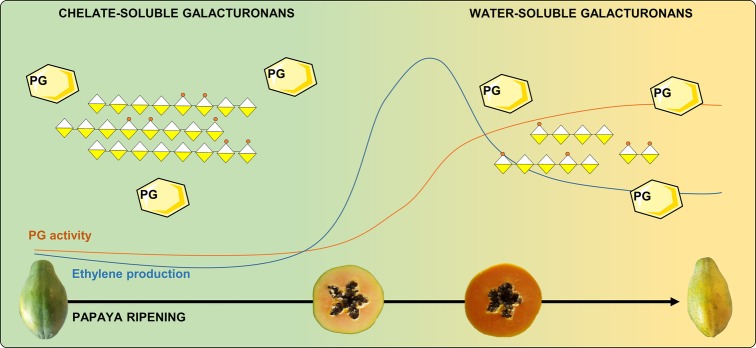
Ethylene production and PG activity during papaya ripening: papaya pectin cell wall solubilization. Ethylene triggers PGs that massively solubilize high-molecular weight pectin by action in the non-methylated areas and releasing the low-molecular weight fractions that will be enriched in methylated fractions due to the lower activity of PME in ripe papayas. PG, polygalacturonase.

Papaya cell wall structural changes during ripening involve pectin with the solubilization of long chains of galacturonans and a decrease in the molecular weight of polysaccharides (Lazan et al., 1995; Manrique and Lajolo, 2004; Shiga et al., 2009). Polygalacturonases act on papaya pulp softening by mobilizing high-molecular weight pectin from less soluble to more soluble cell wall fractions, especially pectin that is tightly bound to cellulose/hemicellulose, and pectins that are bound to each other by calcium bridges (do Prado et al., 2016). Furthermore, the degree of methyl esterification in papaya pectin changes during ripening since unripe papaya pectin has a lower degree of methyl esterification compared to ripe papaya pectin (Manrique and Lajolo, 2002, 2004; do Prado et al., 2017; Prado et al., 2019). This variation during papaya ripening was first associated to higher PME activity (Manrique and Lajolo, 2004). However, no increase in gene expression of PME appears to occur during papaya ripening (Fabi et al., 2010a, 2012, 2014; do Prado et al., 2016), and the activity of PG does not require the simultaneous removal of methyl-esterified groups from pectin (Fabi et al., 2014). Therefore, recent studies support the hypothesis that the increase in the degree of methyl esterification during papaya ripening is a result of the enrichment of the water-soluble pectin fraction that comes from the insoluble fraction due to the massive action of PG rather than an association with increased PME activity (Figure 1; Fabi et al., 2014; do Prado et al., 2016, 2017). The resulted high-methylated low-molecular pectin found in ripe papayas showed anticancer effects in diverse *in vitro* tests (do Prado et al., 2017; Prado et al., 2019).

Although the use of MCP-1 is useful in gaining further insight into the role of ethylene in papaya softening, cold storage is another way to decrease ethylene action after harvesting. This latter approach is useful as a postharvest technique as it decreases fruit ripening rates and, therefore, pulp softening (Gomes et al., 2016). The storage of “Golden” papaya at 10°C for 10 days had been found to be effective in reducing ethylene production and fruit ripening. Notably, after a 10-day cold storage, fruits can be stored at room temperature to restore ethylene production and pulp softening (Gomes et al., 2016). However, when cold storage occurs for a longer period (e.g., 20 days at 11°C), ethylene production did not recover when the fruit was subsequently stored at ambient temperatures (Bron and Jacomino, 2009). It seems that the prolonged inhibition of ethylene, either by the inhibition of receptor sites (1-MCP) or by prolonged storage at low temperatures, strongly affects the recovery of the ethylene-mediated response, which negatively influences the pulp softening that is crucial to the quality of the ripe fruit.

## Pulp Sweetness as a Result of Papaya Primary Metabolism

The qualitative and quantitative composition of primary soluble sugars is crucial to papaya sweetness, although fruit firmness also plays a role as there is a correlation between pulp softening and the perception of sweetness during consumption (Gomez et al., 2002). Thus, it is necessary to understand the key regulatory enzymes involved in the metabolism of soluble sugars, as well as the endogenous and exogenous factors that influence these biochemical pathways, so as to improve both preharvest management and postharvest handling to increase the final sensorial quality of ripe papayas. In papayas, the increment in soluble sugars occurs mainly during fruit growth while still attached to the plant (Zhou and Paull, 2001).

In most fleshy fruits, there are three main enzymes that have a key regulatory role in the accumulation of soluble sugars: sucrose phosphate synthase (SPS), sucrose synthase (SS), and acid invertase (AI; Zhou and Paull, 2001). In papayas, sugar accumulation begins after seed maturation and is accompanied by increased activity of SS during fruit development. Acid invertase also appears to increase throughout papaya development (Zhou and Paull, 2001), and its expression is reduced in harvested unripe papayas. Another increase in AI expression has also been observed after the onset of ethylene production during ripening (Gomez et al., 1999). Sucrose phosphate synthase activity remains low throughout papaya development however (Zhou and Paull, 2001). After harvesting, SPS activity follows the tendency of sucrose formation, since the ratio between SPS activity and sucrose content is constant throughout the papaya ripening process (Gomez et al., 2002). SPS is a highly conserved glycosyltransferase in dicots that catalyzes the transfer of glucose from uridine diphosphate glucose (UDP-Glc) to D-fructose-6-phosphate, thereby forming D-sucrose-6-phosphate (Castleden et al., 2004). As SPS also catalyzes the reversible reaction, it is considered as a key control point of sucrose biosynthesis in both monocots and dicots (Huber and Huber, 1996). Sucrose synthase is also a glycosyltransferase, but it catalyzes the reversible formation of UDP-Glc and d-fructose from UDP and d-sucrose (Zheng et al., 2011). Although SS could act in Glc linked to other nucleotide diphosphate sugars than UDP, such as adenosine diphosphate glucose (ADP-Glc), UDP is the preferred substrate in plants (Kleczkowski et al., 2004). Finally, AI can control the balance between sucrose, glucose, and fructose in fleshy climacteric fruits by an irreversible reaction that cleaves sucrose (Moriguchi et al., 1992).

Climacteric fruits, such as bananas, commonly increase soluble sugars content after harvesting through starch degradation, which directly correlates with pulp sweetening (Shiga et al., 2011; Aquino et al., 2016). Since unripe papayas have low starch content (less than 3% by fresh weight; Oboh et al., 2015), most of the soluble sugars in papayas accumulate during fruit development. However, there is also an increase in sucrose, glucose, and fructose, as well as a pattern of expression and activity of both AI and SPS during ripening (Gomez et al., 1999, 2002). These results suggest a possible role for ethylene-mediated effects on soluble sugar accumulation in ripe papayas. This hypothesis was confirmed by a previous study of our group (Fabi et al., 2007), which demonstrated that 1-MCP-treated papayas have a distinct pattern of sucrose synthesis during ripening compared to untreated papayas. More recently, Shen et al. (2017) showed that other genes related to soluble sugar metabolism, including *UDP-galactose transporter 3* (*UTR3*), *sugar transporter* (*STP*), and *β-fructofuranosidase* (*BFF*), were induced during the ripening of ethylene-treated papaya and reduced in 1-MCP-treated papaya. However, despite ethylene appearing to be important in enhancing *UTR3*, *STP*, and *BFF* expression, it is unknown whether ethylene-induced changes in the expression pattern of these enzymes affect soluble sugar metabolism during ripening and, therefore, the sensorial quality of papaya.

**Figure 2:**
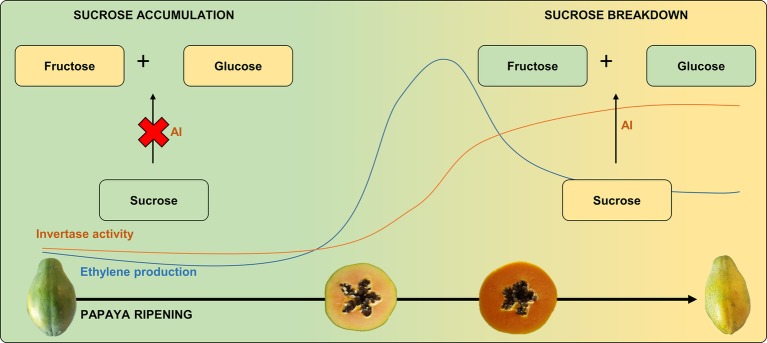
Ethylene production and invertase activity during papaya ripening: papaya sucrose breakdown. Invertase activity is regulated by ethylene burst since sucrose is higher in unripe papayas or in papayas in which ethylene perception is blocked, with a subsequent increase in fructose and glucose after ripening/ethylene production. AI, acid invertase.

During papaya ripening, the sucrose content appears to reduce after the onset of ethylene production, which is in agreement with the increase in AI expression (Gomez et al., 2002; Nogueira et al., 2012). In contrast, 1-MCP-treated papayas have been found to have a 10-fold higher level of sucrose compared to untreated ripe fruit (Fabi et al., 2007). Thus, as AI activity appears to be strongly regulated by ethylene during papaya ripening (Figure 2), exogenous treatments or conditions that affect ethylene production may affect the ratios between sucrose, glucose, and fructose, thereby influencing pulp sweetness.

The use of gamma irradiation in fleshly fruits such as guavas (Zhao et al., 2017) and tomatoes (Guerreiro et al., 2016) could represent an effective method for fruit decontamination, thus reducing postharvest losses (Farkas and Mohácsi-Farkas, 2011). Depending on the intensity of the applied gamma irradiation, the sensorial quality of fruits could be negatively affected because of irradiation-induced changes in fruit metabolism. In papaya, the application of standard irradiation intensities between 0.5 and 1.0 kGy in unripe fruit did not appear to negatively influence fruit ripening (Paull, 1992). However, analysis of fruit metabolism revealed that these gamma irradiation intensities could reduce soluble sugars content in ripe papayas. This reduction appears to be related to a decrease in AI activity, and these changes are associated with reduced ethylene production throughout the ripening of the irradiated fruit (Gomez et al., 1999).

In addition to gamma irradiation, ozone application has been proposed as a method for fruit decontamination. Furthermore, ozone treatment is used to extend shelf life by reducing oxygen concentrations during fruit storage and shipping, thereby delaying the ripening of climacteric fruits (Chrysargyris and Tzortzakis, 2017). Thus, as ozone influences fruit respiration and therefore the onset of ethylene production in climacteric fruits, it is expected that this postharvest treatment will also affect soluble sugar metabolism during papaya ripening. Although a previous study did not report significant differences between the total soluble solid content of ozone-treated and untreated papayas (Bataller et al., 2012), the soluble sugars ratio between sucrose and glucose/fructose in ripe fruits could be altered. Recently, the treatment of unripe papaya with plant extracts, such as Neem (*Azadirachta indica* Juss), has been proposed as an alternative for maintaining food quality for a longer postharvest period (Freitas et al., 2018). However, as with ozone treatment, the observation of fruit quality maintenance for a longer period was not accompanied by an evaluation of soluble sugar metabolism. Therefore, further studies are needed to confirm the effects of ozone as well as other postharvest treatments that may affect ethylene production, since there is a clear role of ethylene on enzymes that orchestrate the metabolism of soluble sugars during papaya ripening.

## Climacteric Alteration of Papaya Flavor

Papayas have a characteristic sweet flavor that has been studied for more than half a century (Katague and Kirch, 1965; Flath and Forrey, 1977). The volatile profile of papaya consists of a mixture of compounds including esters, terpenes, alcohols, and ketones (Pino et al., 2003; Fuggate et al., 2010; Pino, 2014; Jing et al., 2015; Kelebek et al., 2015). Although there is great heterogeneity among the volatile profiles of distinct papaya varieties (Ulrich and Wijawa, 2010; Jing et al., 2015; Kelebek et al., 2015), some compounds appear to be characteristic of the papaya aroma. In this context, linalool and their oxidative derivatives are generally regarded as the main volatile compounds in most of the distinct cultivars of papaya (Devitt et al., 2006; Gomes et al., 2016; Lieb et al., 2018) along with low-molecular weight esters, including ethyl butanoate and methyl butanoate (Almora et al., 2004; Balbontín et al., 2007; Pino, 2014). Considering that the increase in volatile esters is significantly higher in harvested papayas compared to fruit that is still attached to the plant (Fuggate et al., 2010), and considering the magnitude of difference between the volatile profiles of unripe and ripe papayas (Fuggate et al., 2010; Gomes et al., 2016), it appears that ethylene plays an important role in the development of flavor during papaya ripening.

Balbontín et al. (2007) suggested that most of the volatile esters synthesized during papaya ripening are derived from primary and secondary metabolism compounds, such as fatty acids and amino acid. The release of these compounds is stimulated by ethylene treatment (Defilippi et al., 2005; Li et al., 2016). Ethyl acetate, ethyl octanoate, and methyl hexanoate were also found to not be induced in 1-MCP-treated papayas, whereas ethylene-induced papayas increased the amounts of these volatile esters throughout ripening (Balbontín et al., 2007). Interestingly, volatile esters with a higher molecular weight, including butyl hexanoate and octyl acetate, reached higher values in 1-MCP-treated papayas compared to both untreated and ethylene-treated papayas. These results suggests that only the synthesis of the main esters related to aroma quality in ripe papaya—which are those volatile compounds with lower molecular weight produced from C1 and C2 alcohols and C6 and C8 acyl-coenzyme A—were enhanced during the onset of ethylene production (Balbontín et al., 2007).

The volatile profile of ripe papayas also consists of branched-chain volatiles (Rocha et al., 2017; Lieb et al., 2018) derived mainly from the amino acid precursors isoleucine and valine, which are responsible for the formation of ethyl-2-methyl and butyl-2-methyl esters. The synthesis of these branched-chain volatiles also appears to be regulated by ethylene, as 1-MCP-treated papayas have reduced ethyl-2-methyl butanoate levels (Balbontín et al., 2007).

The abovementioned results regarding the synthesis of volatile compounds during ripening provide insights into the development of aroma in ripe papayas. However, little is known about the relationships among the metabolism of these volatile compounds and the sensorial quality of the ripe fruit. In this context, a recent study applied a gas chromatography-olfactometry (GC-O)-assisted approach to optimize the extraction and detection of the main volatile compounds responsible for the aroma of ripe papayas (Rocha et al., 2017). In GC-O, a panel of human assessors describes the aroma of each of the volatile compounds from a sample that has been previously separated through gas chromatography, allowing the identification of the main peaks responsible for the overall aroma of the sample (Brattoli et al., 2011). In summary, GC-O refers to the use of human assessors as sensitive and selective detectors of odor-active compounds (Delahunty et al., 2006), and it is a useful tool to assess the contribution of each volatile compound to a fruit’s aroma. Studies have successfully applied GC-O-assisted approaches or aroma dilution analysis to assess the volatile profile of papayas (Jirovetz et al., 2003; Pino, 2014; Rocha et al., 2017). Jirovetz et al. (2003) and Pino (2014) found linalool as the major compound in papaya flavor. However, the major compounds considered as odor-active and contributors to the typical papaya aroma found in other studies were δ-octalactone (sweet and herbal), benzyl isothiocyanate (papaya), methyl butanoate (fruity), and ethyl butanoate (fruity; Pino, 2014; Rocha et al., 2017).

Gomes et al. (2016) explored the volatile profile of papayas in response to cold storage, which clearly affects ethylene production (Bron and Jacomino, 2009). The authors explored if the cold storage of papayas at temperatures in which the fruit is resistant to cold injury influenced the volatile profile in ripe papayas. The authors found that when papayas were left at 10°C for 10 days and then subsequently at ambient temperature to complete the ripening process, the fruits were able to restore ethylene production, as well as the development of the loss of green color and the increase in pulp softening to a similar extent to that of fruit stored at ambient temperature, but the process was postponed by a few days. However, there were striking differences between the volatile profiles of the two groups. Interestingly, the synthesis of linalool, regarded in GC-O as one of the main volatile compounds in papaya, was affected by cold storage. These reduced linalool levels in cold-stored papayas appeared to be related to the down-regulation of *linalool synthase* (*LIS*) expression (Gomes et al., 2016). Façanha (2016) also found reduced levels of linalool throughout the ripening of 1-MCP-treated papayas and increased levels of this volatile compound in ethylene-treated papayas. Thus, as LIS uses geranyl diphosphate (GPP) to synthesize linalool in a single-step reaction (Gutensohn et al., 2013), the reduced *LIS* expression, and therefore reduced levels of linalool in both cold storage papayas and in 1-MCP-treated papayas, strongly suggests a possible role of ethylene in linalool biosynthesis through modulation of *LIS* expression.

GPP originates from the plastid-localized 2-C-methyl-D-erythritol 4-phosphate (MEP) pathway, which is important not only in the biosynthesis of linalool and other volatile compounds, including β-ionone and 6-methyl-5-hepten-2-one, but also in carotenoid biosynthesis and in the development of the characteristic of pulp color in ripe papayas.

## Pulp Color Changes in Ripening Papayas as a Consequence of Carotenoid Synthesis

The characteristic color of ripe papaya pulp (yellow or orange/red) is due to different types of carotenoids. Carotenoids are molecules with a general structure that consists of a 40-carbon acyclic polyene chain containing 9–11 conjugated double bonds and with or without terminating rings, and they are classified as carotenes (hydrocarbons) or as xanthophylls (oxygenated derivatives; Khoo et al., 2011). Distinct papaya varieties have different pulp colors depending mainly on their carotenoid metabolism during ripening. In general, orange/red varieties have relatively high amounts of lycopene, which is a central compound in the metabolism of carotenoids during papaya ripening and is responsible for the red color not only in papayas (Barreto et al., 2011) but also in tomatoes (Arias et al., 2000), guavas (Rojas-Garbanzo et al., 2017), and watermelons (Perkins-Veazie et al., 2006).

**Figure 3:**
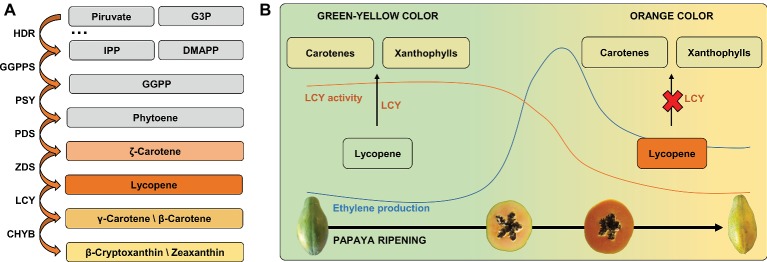
Ethylene production and carotenoids accumulation (LCY activity) during papaya ripening: papaya’s green/yellow color changing to orange/red color. **(A)** Carotenoids derivated from MEP pathway. **(B)** Papaya LCY activity during ripening drives the lycopene accumulation and pulp color changes through the decreased conversion of lycopene in carotenes and xanthophylls. G3P, glyceraldehyde-3-phosphate; IPP, isopentenyl diphosphate; DMAPP, dimethyl allyl diphosphate; GGPP, geranyl-geranyl diphosphate; HDR, 4-hydroxy-3-methylbut-2-enyl diphosphate reductase; GGPPS, geranyl-geranyl diphosphate synthase; PSY, phytoene synthase; PDS, phytoene desaturase; ZDS, ζ-carotene desaturase; LCY, lycopene cyclase; CHYB, carotene hydroxylase.

Most of the over 600 naturally occurring carotenoids (Sigurdson et al., 2017) originate from the MEP pathway (Figure 3A), which starts with a reaction between pyruvate and glyceraldehyde-3-phosphate, resulting in the downstream production of isopentenyl diphosphate (IPP) and dimethyl allyl diphosphate (DMAPP; Ruiz-Sola and Rodríguez-Concepción, 2012; Yang and Guo, 2014). Then, three IPP molecules and one DMAPP molecule are used as substrates by geranyl-geranyl diphosphate (GGPP) synthase for the synthesis of GGPP, a 20-carbon molecule (Majer et al., 2017). In addition to the presence of relatively high levels of lycopene, orange/red papayas present lower amounts of carotenoids that are synthesized downstream to lycopene in the MEP pathway, such as β-carotene, β-cryptoxanthin, and zeaxanthin (Schweiggert et al., 2011). For both papaya cultivars “Golden” and “Sunrise Solo,” all-trans-lycopene was the main carotenoid in early stages and all-trans-β-cryptoxanthin was the main carotenoid in overripe fruits (Martins et al., 2016).

Yellow pulp varieties are characterized by the presence of these last carotenoids with very low to no detectable levels of lycopene (Shen et al., 2019). As the metabolism of papaya carotenoids starts from phytoene and occurs in a well-known cascade process (Figure 3B), it is possible to establish a relationship between the pattern of enzymes that acts downstream to phytoene and the color of papaya pulp during ripening. Geranyl-geranyl diphosphate is the precursor of chlorophylls, ubiquinones, and tocopherols. Phytoene synthase (PSY) uses two molecules of GGPP to produce phytoene, a colorless 40-carbon acyclic polyene molecule, which is the first step in carotenoid biosynthesis in the MEP pathway. Phytoene can be further used as a substrate by phytoene desaturase (PDS) to produce ζ-carotene, which can be a substrate for ζ-carotene desaturase (ZDS) for the synthesis of lycopene, a bright red carotenoid widely found in the pulp of orange/red papaya (Nisar et al., 2015). In yellow papayas, there is no significant accumulation of lycopene because of the conversion of phytoene by PDS and ZDS and by both lycopene β-cyclases (LCY-β) and carotene hydroxylases (CHYB). These enzymes rapidly convert lycopene into xanthophylls and β-carotene (Blas et al., 2010; Shen et al., 2019). In orange/red papayas, the initial stages of ripening are characterized mainly by the presence of xanthophylls, including β-cryptoxanthin, which are synthesized from lycopene downstream by lycopene β-cyclase (LCY-β; Blas et al., 2010; Schweiggert et al., 2011). However, after the onset of ethylene production in red/orange papayas, the conversion of lycopene into cyclic carotenoids appears to be strongly decreased due to lycopene accumulation in pulp (Barreto et al., 2011; Shen et al., 2019). The accumulation of lycopene in orange/red papayas compared to yellow papayas seems to occur both by a frame shift mutation in the *LCY-β2* gene, which results in a dysfunctional enzyme phenotype, and by other LCY genes (e.g., *LCY-β* and *LCY-ε*) that are down-regulated during orange/red papaya ripening (Shen et al., 2017). The ζ-carotene desaturase enzyme responsible for converting phytoene into lycopene shows a different pattern of expression during ripening and also between the cultivars “Golden” and “Sunrise Solo,” while the lycopene β-cyclase gene, responsible for converting lycopene to β-carotene, is up-regulated in both cultivars (Martins et al., 2016).

Interestingly, both ethylene- and 1-MCP-treated papayas had lower levels of minor carotenoids as compared to those of untreated papaya, similar to what was previously reported for the major carotenoids (Fabi et al., 2007; Barreto et al., 2011). Furthermore, the treatment of distinct papaya varieties with 1-MCP significantly reduced the carotenoid content in fruit pulp throughout ripening (Moya-León et al., 2004; Fabi et al., 2007; Barreto et al., 2011). Barreto et al. (2011) suggested that the impairment on carotenoid accumulation in papaya pulp by 1-MCP could occur either by the consumption of early carotenoid precursors including GGPP, or by inhibiting PSY or PDS activity. The latter hypothesis was confirmed by Fu et al. (2016), who revealed that a transcription factor (CpNAC1) induced by ethylene enhances the expression of PDS genes (e.g., *CpPDS2* and *CpPDS4*). Recently, Fu et al. (2017) provided new insights into the role of other transcription factors that regulate ethylene responses and are involved in the regulation of several genes related to carotenoid biosynthesis. Therefore, as with pulp softening, sweetness, and the development of flavor, the carotenoid content in papayas is also regulated by ethylene-mediated responses during fruit ripening. Thus, while further studies are needed to define the specific genes whose expression relates to changes in the carotenoid content in papaya pulp, it is known that the reduction of ethylene production at low temperatures influences the composition of carotenoids in ripe papaya pulp (Rivera-Pastrana et al., 2010).

## Conclusions

Changes in the primary and secondary metabolism of papaya are mainly dependent on ethylene, whose onset burst occurs 2–3 days after the harvest of unripe fruit. Ethylene-triggered events during papaya ripening include an increase in PG and AI expression that are related to pulp softening and sweetening, respectively, as well as changes in carotenoid metabolism that influence both aroma and color, thereby leading to the formation of the expected quality attributes in ripe papaya. As ethylene-triggered events clearly affect the final quality of ripe papayas, studies have investigated the regulatory mechanisms that regulate ethylene function in papaya. Despite recent findings that highlight the ethylene-triggered events during papaya ripening, more efforts are needed to fully understand the key downstream regulators of ethylene in papaya pulp to better develop pre- and postharvest practices to extend papaya shelf life without resulting in losses in quality and nutritional aspects.

## Author Contributions

SP provided illustrations. Both SP and JF wrote the manuscript, contributed to critical review of the manuscript, and revised and approved the manuscript.

### Conflict of Interest Statement

The authors declare that the research was conducted in the absence of any commercial or financial relationships that could be construed as a potential conflict of interest.
